# Optical, contact-free assessment of brain tissue stiffness and neurodegeneration

**DOI:** 10.1364/BOE.545580

**Published:** 2025-01-06

**Authors:** Philip Binner, Ilya Starshynov, Gonzalo Tejeda, Aisling McFall, Colin Molloy, Giuseppe Ciccone, Matthew Walker, Massimo Vassalli, Andrew B. Tobin, Daniele Faccio

**Affiliations:** 1 School of Physics and Astronomy, University of Glasgow, Glasgow, United Kingdom; 2 School of Molecular Biosciences, University of Glasgow, Glasgow, United Kingdom; 3 Institute for Bioengineering of Catalonia (IBEC), the Barcelona Institute for Science and Technology (BIST) Barcelona, Spain; 4 James Watt School of Engineering, University of Glasgow, Glasgow, United Kingdom

## Abstract

Dementia affects a large proportion of the world’s population. Approaches that allow for early disease detection and non-invasive monitoring of disease progression are desperately needed. Current approaches are centred on costly imaging technologies such as positron emission tomography and magnetic resonance imaging. We propose an alternative approach to assess neurodegeneration based on diffuse correlation spectroscopy (DCS), a remote and optical sensing technique. We employ this approach to assess neurodegeneration in mouse brains from healthy animals and those with prion disease. We find a statistically significant difference in the optical speckle decorrelation times between prion-diseased and healthy animals. We directly calibrated our DCS technique using hydrogel samples of varying Young’s modulus, indicating that we can optically measure changes in the brain tissue stiffness in the order of 60 Pa (corresponding to a 1 s change in speckle decorrelation time). DCS holds promise for contact-free assessment of tissue stiffness alteration due to neurodegeneration, with a similar sensitivity to contact-based (e.g. nanoindentation) approaches.

## 1. Introduction

Neurodegeneration is a widespread disease, with an estimated 50 million people living with dementia worldwide and a prediction of 130 million people by 2025 [1]. A key aspect limiting neurodegenerative research and assessment is a lack of diagnostic tools [2].

Neurodegeneration and ageing (with related cognitive decline) have been linked to changes in the mechanical properties of the brain [3–7] and remote or non-invasive methods that can inform about these mechanical properties hold great diagnostic promise. Magnetic resonance elastography (MRE) is the gold standard approach for the non-invasive assessment of tissue stiffness and viscosity and has shown that brains affected by neurodegeneration are softer than their healthy counterparts in both animal models and human patients [7–20]. In vivo MRE measurements on neurodegenerated mouse brains indicate percentage differences in healthy and diseased tissue elastic moduli ranging from 2% up to 35% [8]. Furthermore, postmortem neurodegenerated brains also showed signs of spongiosis, a conformational change of the brain structure induced by neurodegeneration [21]. However, current methods to quantify neurodegeneration come with drawbacks: symptomatic diagnoses [22,23] are subjective to the examiner, histology [24] can require complex fixing and staining procedures, and magnetic resonance imaging [18,25] is generally costly and subject to limited availability.

Optical methods offer a possible solution as they can be quantitative, label-free, low-cost, and potentially applied both ex vivo and in vivo. Diffuse correlation spectroscopy (DCS) is one cost-effective technique that is typically simple in design. In our paper, we use DCS for the remote sensing of neurodegeneration in ex vivo mouse brain samples.

DCS is a scattered light technique that has been applied to the remote, non-invasive study of biological matter and is currently popular for brain sensing and imaging applications, taking advantage of near-infrared light’s sensitivity to red blood cell motion [26–33]. It probes the motion of scatterers inside a medium, such as the Brownian motion or random ballistic flow [34–39]. Scatterer motion is also related to a material’s mechanical properties, such as elasticity and viscosity [40]. Thus, the key DCS parameter, the decorrelation time $\tau_{c}$ depends also on a material’s mechanical properties and has been shown to linearly correlate with a medium’s storage and loss modulus. This has been demonstrated in nonbiological media, such as hydrogel samples [41–44] and has also been applied to biological media, such as the identification of breast tumours [45,46].

In this work, we show that DCS can be used to assess the mechanical stiffness of brain tissue in mouse models. We first calibrate the technique using hydrogel samples and then show that DCS can differentiate between prion disease (a mouse-model proxy for Alzheimer’s disease in humans) and healthy brain samples ex vivo. Importantly, DCS is a fast, inexpensive and non-invasive technique that does not require contact with the sample, which potentially could be scaled towards in vivo applications in the future, given its current application for measuring cerebral blood flow in vivo and in humans.

However, one limitation of DCS is its long acquisition time required, where one must statistically average many independent speckle modes to correctly estimate the speckle decorrelation time [47]. This is not so much a problem for fast blood flow measurements but is for tissue measurements, where dynamics are on the timescales of seconds. In the past, multispeckle methods have been incorporated with photodiode measurements [48]. More generally these days, multipixel detectors are used here, where the speckle size is set equal to a pixel to maximise the number of speckle modes averaged. Nonetheless, the problem may still persist, and in our paper, we address it with a simple approach that we call the pixel-stacking method.

## 2. DCS experimental setup

**Fig. 1.**
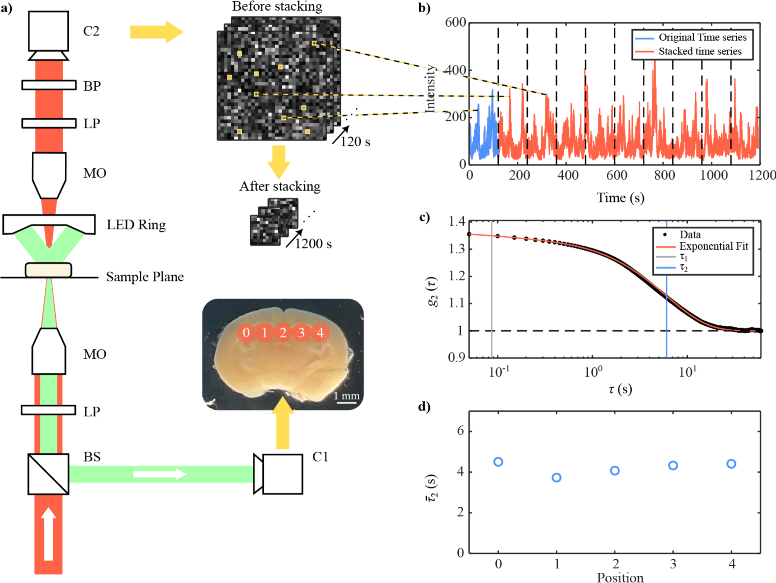
**Experimental layout and workflow.** a) A schematic diagram of the DCS apparatus. 633 CW nm light is passed through a linear polariser (LP) focused onto a 1 mm diameter spot on the mouse brain hippocampus with a microscope objective (MO). The transmitted light is cross-polarised, filtered with a 633 nm bandpass filter (BP) and imaged with a single-photon camera (C2). The bottom surface of the brain slice is also imaged for reference with a CMOS camera (C1) and green LED light source. An example white light image of a typical brain slice is shown as an inset, which shows a 5-point scan along the hippocampus of the brain slice. Another inset shows the optical speckle pattern measured by C2. The pixels of this speckle pattern are concatenated to effectively increase the acquisition time of the experiment. 10 random pixels in the speckle series are concatenated to make a lower-resolution speckle that is 10 times its original length. b) The time series of a single pixel is shown in blue, and the whole stacked time series in red. black dashed vertical lines are shown where time series have been concatenated. d) An intensity correlation ( $g_{2}$ ) trace and corresponding multi-exposure fit. Vertical lines show the $\tau_{1}$ and $\tau_{2}$ values for this fit. d) $\bar{\tau_{2}}$ measurements for all positions scanned in a single brain sample. These measurements are later used for statistical analysis.

A schematic diagram of the DCS apparatus is shown in Fig. 1(a). A 633 nm continuous-wave laser (RPMC RO633) was focused into a 1 mm-diameter spot on the sample surface, and a single-photon avalanche diode (SPAD) camera (PhotonForce PF32) captured the transmitted optical speckle pattern. Images of the speckle pattern were acquired for 2 minutes at 100 fps. Brain slices were chosen for measurement, which made it easier to collect light scattered through the brain’s hippocampus. Moreover, measurements were carried out in transmission so as to maximise the collection of multiply scattered light, without any glare from reflected light. The sample’s incoming and outgoing laser light were cross-polarised to limit transmitted ballistic light, and a bandpass filter blocked background light from reaching the SPAD camera. A translation stage was used to perform a five-point scan along the hippocampal regions of the brain slice. This scan was repeated three times for each brain sample. Thus, each brain sample has three repeated scans and five acquisition positions, totalling fifteen measurements per brain sample hippocampus. A reference camera (Basler acA2440-75uc) imaged the bottom surface of the brain sample and helped focus the laser onto the brain’s hippocampus. The reference camera and its corresponding LED light source were set up in a dark-field microscopy arrangement for better contrast. The speckle size was controlled by a microscope objective placed after the sample plane. The objective’s distance to the sample surface was set so that the SPAD camera imaged one speckle per pixel, which has been shown to improve the signal-to-noise ratio of the DCS measurement [31,39]. This calibration was done using a spatial autocorrelation [49] and an intermediary sCMOS camera. Lastly, before each DCS scan, we verified with a temperature probe (Thorlabs TSP01) that the temperature of the brain had acclimatised to the room as to limit any potential effects of temperature on the DCS measurement.

## 3. DCS data analysis

A key parameter in DCS measurement is $\tau_{c}$ , which measures how quickly the speckle pattern produced by multiple light scattering in a medium changes. The faster the motion of the medium, the faster the speckle pattern changes, and the shorter the $\tau_{c}$ that is measured. DCS uses the normalised intensity autocorrelation function to find $\tau_{c}$ , which is defined as,

(1) $$g_{2}(\tau )=\frac{\left\langle I(t)I(t+\tau )\right\rangle }{\langle I(t)\rangle^{2}},$$

where T is the total acquisition time, I(t) and I(t+τ) represent the intensity at a given pixel at times t and t+τ and ⟨⋅⟩ denote a time average. The autocorrelation is normalised by the mean pixel intensity squared, such that a full decorrelation of the signal corresponds to $g_{2}(\tau )=1$ and is typically reached for acquisition times much greater than $\tau_{c}$ , e.g. for $∼100\tau_{c}$ . More explicitly, the Wiener-Khinchin theorem allows to calculate the autocorrelation as an inverse Fourier transform of $|I{|}^{2}$ as a function of frequency $f_{t}$ , i.e. $g_{2}(\tau )=F^{-1}\{|I(f_{t}){|}^{2}\}/{\left[(1/T)\int_{0}^{T}I(t)dt\right]}^{2}$ .

Once the individual $g_{2}$ traces are obtained for each pixel on the camera, they can be averaged together to form a mean $g_{2}$ trace, thus significantly improving the signal-to-noise ratio [31,50,51]. There are many ways to obtain $\tau_{c}$ from the $g_{2}$ decorrelation trace. In the past, indexing approaches and single exponential fits have been used [31,39,52]. The fitting approach provides further information on the sample’s properties. For example, a diffusive fit may be applied to a sample whose scatterers undergo Brownian motion, whereas a ballistic fit may be used more for blood flow applications [39]. In some cases, single exponentials do not fit the decay of the medium well. For example, when scatterers move according to different time scales, a multi-exponential fit with multiple decorrelation time components is more applicable. A recent study on live mice by Liu et al. [53] used the below multi-exponential fit, which we have found works best for our data,

(2) $$\begin{array}{r} g_{2}(\tau )=1+\beta [\rho_{1}|\exp(-\tau /\tau_{1})|+\rho_{2}|\exp(-\tau /\tau_{2})|\\ +(1-\rho_{1}-\rho_{2})]. \end{array}$$

Here, β is a contrast term and $\rho_{1}$ and $\rho_{2}$ represent the average fraction of slow and fast-moving scatterers, respectively. The fit splits $\tau_{c}$ into both a fast $\tau_{1}$ , a slow $\tau_{2}$ component, and also incorporates a static $\left(1-\rho_{1}-\rho_{2}\right)$ component. In Liu et al.’s example, the fast component may describe blood flow, and the slow component may describe intracellular motility. However, the fast component may be used to describe any form of fast decorrelating element in an experiment, such as apparatus vibration.

Fig. 1 also illustrates the post-processing pipeline. Optical coherence tomography studies have had similar problems to DCS in estimating $\tau_{c}$ for short acquisitions, and we have developed our analysis from [54] to reduce the total acquisition time of our experiment. Here, we concatenate random pixels of the speckle series, as shown in the upper inset of Fig. 1(a), effectively increasing the acquisition time of a time series. This stacking procedure allows us to measure $\tau_{c}$ for acquisition times equivalent to $10\tau_{c}$ rather than $100\tau_{c}$ . Specifically, for each acquisition, we reshape the 1000 pixel time series of acquisition time 120 s into the 100 pixel time series of effective acquisition time 1200 s by randomly concatenating the 10 pixel time series. The stacked time series is shown in Fig. 1(b). After the stacking procedure, the mean $g_{2}$ trace was calculated for the remaining concatenated pixels in the speckle pattern. Then, the multi-exponential fit above was applied to extract the slow $\tau_{2}$ component for each scan point of each brain sample. The fast $\tau_{1}$ component was on the scale of milliseconds and was deemed to account for experimental noise. On the other hand, the $\tau_{2}$ component had a $\rho_{2}>>\rho_{1}$ , i.e., the dominant signal and was used for analysis. An example $g_{2}$ trace with its multi-exponential fit are shown in Fig. 1(c). Lastly, for each brain sample, we scan and obtain 5 spatial measurements across its hippocampus, which are shown in Fig. 1(d). This 5-point scan is repeated 3 times and averaged.

Lastly, the optical density OD of the brain slices was measured using a power meter (Thorlabs PM400 with S120C head). The OD of a material describes how scattering that material is and thus influences the DCS measurement. For example, a high OD brain section may exhibit more light scattering and therefore a smaller $\tau_{c}$ than a lower OD section. To ensure that OD was not influencing the DCS measurement, we later performed statistical tests that showed no difference in OD between the experiment’s brain slice populations. This comparison of $\tau_{c}$ and OD is sufficient for our case, where we study light predominantly scattered from the hippocampus, but is not suitable, for example, for whole brains, where the structure is more complex.

## 4. Mouse brain samples

We have published previously that murine prion disease shows many of the molecular hallmarks of Alzheimer’s disease (AD), including the accumulation and spread of disease-causing misfolded protein, neuroinflammation and an up-regulation of protein markers of AD, for example, Apolipoprotein E [55–57]. Furthermore, mouse prion disease is progressive, where early phases are associated with synaptic loss and neuroinflammation that results in deficits in learning and memory and ultimately in death [55,58,59]. In this way, murine prion disease is considered a model that represents many aspects of human neurodegenerative diseases such as AD, Parkinson’s disease and Huntington’s disease that start with the aggregation of a misfolded seed particle [60,61]. Such diseases spread in a “prion-like” manner [58,62] throughout the brain resulting in neuronal loss and a change in brain architecture that we aim to detect using DCS. All animal work was performed under a project license in agreement with U.K. Home Office Regulations. Three mouse populations were used for our study: 1) a non-diseased wildtype group (labelled as control-WT), 2) a non-diseased control tg37 group (tg37 hemizygous mice express three times the cellular prion protein content than WT mice, labelled as control-tg37) and, 3) a prion-diseased tg37 group (labelled as prion-tg37). At 3-4 weeks of age, prion-tg37 mice were inoculated with 1% Rocky Mountain Laboratory prion-infected brain homogenate via intracerebral injection. The control-tg37 mice were instead inoculated with normal brain homogenate. Prion-tg37 mice were examined daily for indicators of scrapie prion disease as previously described in [57], and animals were culled from 11-13 weeks post-inoculation when they developed clinical signs of terminal disease. Overall, 48 mice were used, consisting of 10 control-WT, 21 control-tg37, and 17 prion-tg37. Since for DCS measurements, $\tau_{c}$ depends on sample thickness [63,64], each brain sample was cut precisely to 1 mm using a vibratome (Leica VT 1200S). After cutting, samples were kept in an oxygenated artificial cerebrospinal fluid buffer solution to preserve the brain for as long as possible. Brain cross-sections with larger hippocampal regions, where misfolded prion proteins typically aggregate [55], were then chosen for the DCS measurement.

## 5. Confirmation of neurodegenerative disease through Western blots

**Fig. 2.**
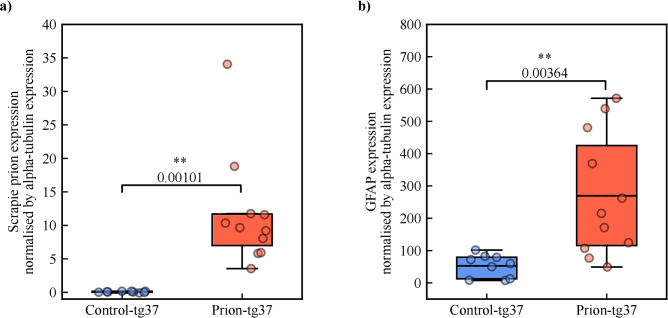
**Confirmation of prion disease.** a) Scrapie prion expression Western blot results with Welch’s t-test p-value annotated. b) The GFAP expression, also with Welch’s t-test analysis. The scrapie prion and GFAP expressions have been normalised by that of alpha-tubulin, a loading control. Both p-values indicate statistical significance and confirmation of neurodegeneration. Each box represents the interquartile range (IQR) centred around the median of each population. Whiskers represent 1.5× IQR. Solid horizontal lines represent a mean value. Each point represents a different brain sample. * and ** are given for p-values less than 0.05 and 0.01, respectively.

To confirm that the prion-tg37 group was diseased with a gold standard method, we performed Western blots on a subset of the mouse brain samples (9 control- and 11 prion-tg37). Here, expression of the misfolded scrapie prion and glial fibrillary acidic protein (GFAP) are prion disease markers [56,57,65]. The samples were digested in proteinase-K (PK) to determine the level of scrapie prion expression and non-digested otherwise to find the expression of GFAP and alpha-tubulin. Alpha-tubulin was used as a loading control to compare various Western blots. Figure 2(a-b) presents the scrapie prion and GFAP expression for the control- and prion-tg37 populations. Welch’s t-test shows statistical significance between the two populations for both scrapie prion and GFAP expression, thus confirming that the prion-tg37 population is diseased.

## 6. Nanoindentation measurements

Past work has linked $\tau_{c}$ to mechanical measurements over a range of synthetic samples from agarose hydrogels to polydimethylsiloxane elastomer samples [41]. To show that our device performs similarly, we also calibrate our setup using synthetic samples made of polyacrylamide hydrogels with varying and controllable stiffness before performing DCS measurements. Hydrogels were prepared according to recipes in [66]. Specifically, four hydrogel samples were made with the expected average Young’s modulus (stiffness) E=200±30 , 480±160 , 710±240 , and 1100±340 Pa, which were chosen with respect to the brain nanoindentation measurements. ${\mathrm{TiO}}_{2}$ at a final 5 mg/mL concentration was added to the pre-polymer solutions to induce light scattering for DCS measurements. This concentration of ${\mathrm{TiO}}_{2}$ produced hydrogels of a similar optical density (OD) as the mouse brain slices ( $OD_{hydrogels}=1.80\pm 0.01$ , $OD_{mousebrains}=1.87\pm 0.01$ ). Past rheology measurements of neurodegenerated brain tissue composed of shear modulus measurements [7–20]. Mechanical shear rheometers often use large millimetre-sized probes, so we opted for a nanoindentation device to perform rheology on our brain slices. Nanoindentation devices measure a sample’s Young’s modulus, which is linearly related to its shear modulus through the equation E=2G(1+ν)∼3G , for the Poisson ratio of the brain ν∼0.5 [67,68]. Nanoindentation measurements were conducted using a fibre-optic-based nanoindentation device (Chiaro, Optics 11 Life) mounted on top of an inverted optical microscope (Axiovert 200M, Zeiss), following a standardised protocol [69]. A cantilever with spring constant k=0.022 N/m equipped with a spherical bead of radius R=3μ m was used. Force-distance F−z curves were acquired by moving the probe at a constant speed of 2 μ m/s over a vertical range of 12 μ m, resulting in an indentation depth of ∼ 6 μ m. The Young’s modulus was extracted by fitting the approach segment of F−z curves with the Hertz model up to an indentation depth defined by 60 % of the maximum load. Only curves with a clear out-of-contact region followed by an in-contact region were analysed.

**Fig. 3.**
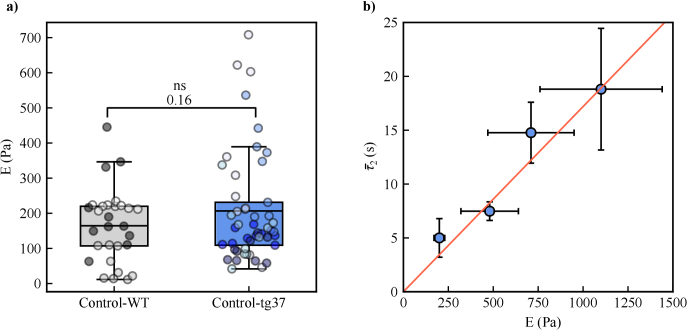
**DCS and nanoindenter calibration measurements.** a) Box plots showing Young’s modulus E , nanoindentation measurements for both control-WT and control-tg37 (sample sizes of 29 and 49 respectively) groups and Welch’s t-test analysis (performed with Scipy’s *ttest_ind* function) showing no significance (ns) between the two control populations’ E . Different coloured data points represent a different brain slice. Box and whiskers are as previously defined. b) DCS $\tau_{2}$ decorrelation time vs E for hydrogel samples (8 DCS measurements per sample) with a linear fit (solid line, r-squared =0.9369 ). Error bars represent the standard deviation.

Figure 3(a) shows box plots of Young’s modulus E measurements annotated with Welch’s t-test comparisons for the two control groups of the experiment. No significance (ns) was observed between the two control strains of mice by nanoindentation, where the E of control-WT and control-tg37 groups measured 164.51±19.24 and 206.70±22.19 Pa, respectively, confirming that there is no meaningful difference in brain tissue stiffness between the wild-type and control mouse models. Nanoindentation measurements could not be performed on the prion-diseased samples, as 2M NaOH is required to denature misfolded prion protein and clean the nanoindenter device, a treatment that would also have permanently damaged the measurement device.

Figure 3(b) presents the $\tau_{2}$ measurements of the hydrogel samples and shows a linear dependence between their $\tau_{2}$ measurement and Young’s modulus ( $\tau_{2}=0.017E$ ) that is in line with the findings of [41] and indicates, for our DCS setup, a sensitivity of ∼60 Pa/second (i.e. a change in DCS $\tau_{2}$ of 1 second corresponds to a change in stiffness of 60 Pa). The results show that the DCS apparatus is linearly correlated to stiffness and validates the DCS technique for the following experiment measuring $\tau_{2}$ of healthy and neurodegenerated brain samples. We note that the lack of diseased nanoindentation measurements is a limitation of our work. However, the hydrogel samples were made so as to have the same range of OD and stiffness compared to the brain section hippocampi and suggest that brain $\tau_{c}$ values determined by DCS should reflect the linear trend shown in Fig. 3(b).

## 7. Optical density measurements between healthy and neurodegenerated brain samples

**Fig. 4.**
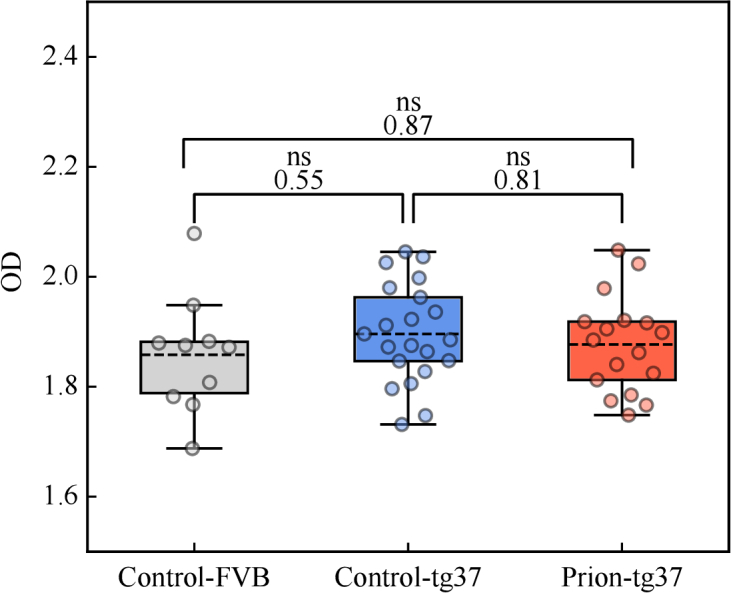
**OD results.** Box plots of OD for control-FVB, control-tg37, and prion-tg37 populations with Welch’s t-test annotations. No statistical significance was observed indicating that changes in the brain’s opacity is not a marker for neurodegeneration. Each circle is a mean OD measurement of a single brain sample. Box and whiskers are as previously defined.

The OD measurements for each brain slice population are shown in Fig. 4. OD measurements presented are mean measurements around the brain’s hippocampus. Because these measurements were done with a different apparatus and with less control than the above DCS measurements, we quote a mean OD value rather than an OD value for each scan point, as in the DCS results. One-way analysis of variance (ANOVA) post hoc Tukey’s test p-values are annotated in the figure and indicate no statistical significance between the mouse strains in terms of OD, therefore showing that DCS is the more sensitive to neurodegeneration-associated changes out of the two optical methods.

## 8. Statistical differences between healthy and neurodegenerated brain DCS measurements

**Fig. 5.**
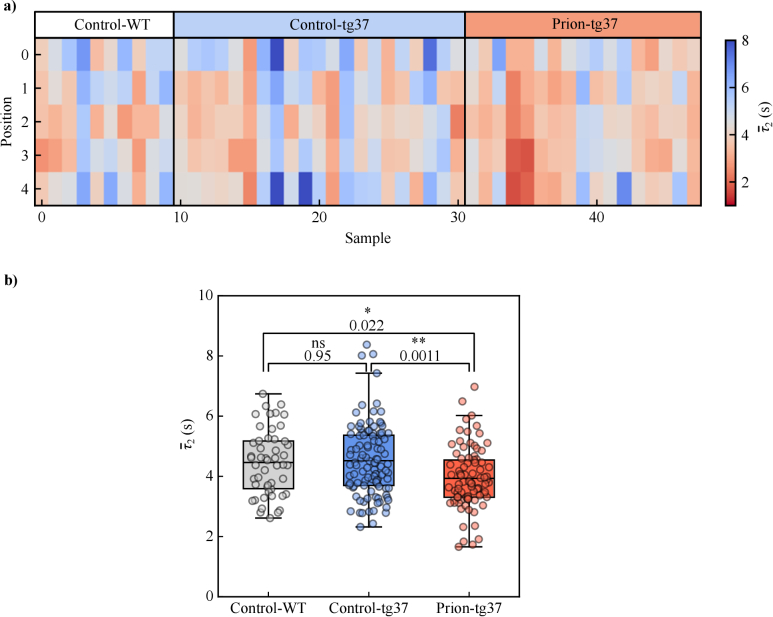
**DCS results.** a) Mean $\tau_{2}$ colourmaps of the control and diseased populations for each sample and scan position. Boundaries indicate which samples belonged to which population. b) $\bar{\tau_{2}}$ box plots of control and diseased populations. A one-way ANOVA with Tukey’s multiple comparisons tests was performed across each population, where Tukey’s test p-values are annotated between respective populations. Individual $\bar{\tau_{2}}$ data points are overlaid over the box plots. Box and whiskers are as previously defined.

Figure 5(a) shows colourmaps of mean $\tau_{2}$ values for each sample and acquisition position, labelled 0-4 (see also inset in Fig. 1). Samples from each control-WT, control-tg37, and prion-tg37 population are grouped together for illustrative purposes but were measured blindly. An average pattern can be seen by eye with the prion-tg37 samples measuring shorter $\tau_{2}$ than the control-tg37 and control-WT samples, although, of course, the statistical relevance of this difference is to be verified quantitatively. Box plots of control-WT, control-tg37, and prion-tg37 are presented in Fig. 5(b) and mean $\tau_{2}$ measurements for each population are shown in Table 1. There is a 13.05% difference between the Control-tg37 and Prion-tg37 $\tau_{c}$ measurements, which is comparable to the sensitivity of mouse model MRE measurements of [10–12,70].

**Table 1.** Mean and standard error of $\tau_{2}$ measurements for each mouse strain.

	$\bar{\tau_{2}}$ (s)
Control-WT	4.46 ± 0.15
Control-tg37	4.52 ± 0.12
Prion-tg37	3.93 ± 0.11

ANOVA was first performed on the $\tau_{2}$ measurements across all three populations and gave a p-value of 0.0009. Tukey’s post-hoc analysis was then performed to verify which population means differed. Tukey’s test p-values are annotated between each corresponding box plot in Fig. 5(b). We saw no statistical significance in $\tau_{2}$ when considering both the control-WT and control-tg37 populations, in agreement with the previous nanoindenter E measurements (Fig. 3). However, a statistically significant difference is seen between the prion-tg37 population and both control populations, which is supported by our previous Western blot analysis (Fig. 2). This shows a statistically relevant difference between the control and prion-tg37 model mechanical tissue properties as probed by DCS, which is indeed due to the progression of neurodegeneration.

## 9. Conclusion and future prospects

We show that DCS can differentiate between healthy and prion-diseased brain samples on a statistical basis, with prion-diseased brain samples exhibiting a shorter $\tau_{2}$ than healthy brain samples, which suggests that these diseased samples have a lower stiffness.

Aside from DCS, other techniques that can probe mechanical properties also exist, such as Brillouin microscopy [71,72], optical coherence elastography (OCE) [73–75] and laser speckle contrast imaging (LSCI) [76–78]. Although, Brillouin microscopy can produce detailed mechanical property maps of a sample, it is not well-suited to macroscopic applications and therefore also scaling it towards deep applications will be difficult in a non-invasive manner. OCE applies mechanical stress to a sample, for example, through an ultrasound transducer, and optically measures the medium’s response to obtain information about its mechanical properties. Deep applications of OCE are challenging, where needle probes have previously been used in this respect [73] and require the skull to be removed [79]. DCS on the other hand is already being used in in vivo human applications, albeit for blood flow, and is therefore a promising technology to develop further in this respect. DCS has also recently been incorporated with acoustic stimulation to improve its spatial resolution [80], however, it is not currently tested for stiffness measurements in this respect. Like OCE, LSCI is a speckle-based technique similar to DCS that has also been used in conjunction with acoustic stimulation of the sample [76–78]. Building from LSCI, however, there have been recent developments in speckle contrast optical spectroscopy that show sensitivity to tissue mechanics, such as intracellular motility, when source-detector separations are short without the need for acoustic stimulation [53]. This technology has been applied to live mice and could therefore be a promising alternative to DCS.

Although we extract a statistical difference between the control and prion populations we realise that a significant number of brain samples is required. For this technique to become medically relevant, we should aim towards differentiating healthy and diseased brains on a sample-by-sample basis. Neurodegeneration is a slow and irreversible process, so future work should focus on longitudinal studies to evaluate the possibility of replacing the statistical properties across different mice with the temporal statistical properties of the same mouse. This would require fewer samples, as we could see how the brain of a subject with neurodegeneration changes as the neurodegenerative disease progresses. However, the measurements will also need to be translated into in vivo tests and our setup adapted into a reflection scheme. DCS has been mainly applied to blood flow measurements in the past, and work to distinguish a signal from tissue mechanics when there is also blood flow has not been reported on. In this case, one realistic next step of this experiment could be to perform live measurements that utilise either surgical windows in the skull [81,82] or non-harmful skin clearing methods [83] alongside DCS for live and longitudinal mouse model neurodegeneration measurements. Moreover, for whole brain and in vivo measurements, it may be necessary to distinguish between different brain regions, in which case one must have a depth-resolved measurement of $\tau_{c}$ . Important work in time-domain DCS is being established, which gives DCS depth sensitivity [27–85]. Additionally, optical transparency regions in biological media also present an opportunity to achieve depth sensitivity if infrared wavelengths of input light are used [86,87].

## Supplemental information

Supplement 1 Raw Western blot figure https://doi.org/10.6084/m9.figshare.28067636

## Data Availability

Data underlying the results presented in this paper are available at [88].

## Supplemental document

See Supplement 1 for supporting content.
