# Supplementary material for: Optical, contact-free assessment of brain tissue stiffness and neurodegeneration
Source: Biomed Opt Express. 2025 Jan 6;16(2):447–59. doi: 10.1364/BOE.545580 (PMC11828460; doi:10.1364/BOE.545580)
Supplement: Supplement 1 [file boe-16-2-447-s001.pdf]

Supplemental Document

Biomedical Optics EXPRESS

Optical, contact-free assessment of brain tissue stiffness and neurodegeneration: supplement

PHILIP BINNER,[1] ILYA STARSHYNOV,[1] GONZALO TEJEDA,[2] AISLING MCFALL,[2] COLIN MOLLOY,[2] GIUSEPPE CICCONE,[3,4] MATTHEW WALKER,[4] MASSIMO VASSALLI,[4] ANDREW B. TOBIN,[2] AND DANIELE FACCIO[1,*]

[1] *School of Physics and Astronomy, University of Glasgow, Glasgow, United Kingdom*
[2] *School of Molecular Biosciences, University of Glasgow, Glasgow, United Kingdom*
[3] *Institute for Bioengineering of Catalonia (IBEC), the Barcelona Institute for Science and Technology (BIST) Barcelona, Spain*
[4] *James Watt School of Engineering, University of Glasgow, Glasgow, United Kingdom*
[*] *daniele.faccio@glasgow.ac.uk*

This supplement published with Optica Publishing Group on 6 January 2025 by The Authors under the terms of the Creative Commons Attribution 4.0 License in the format provided by the authors and unedited. Further distribution of this work must maintain attribution to the author(s) and the published article's title, journal citation, and DOI.

Supplement DOI: https://doi.org/10.6084/m9.figshare.28067636

Parent Article DOI: https://doi.org/10.1364/BOE.545580

Optical, contact-free assessment of brain tissue stiffness and neurodegeneration: supplemental document

1. RAW WESTERN BLOTS

The raw western blots corresponding to Fig. 2. are shown in Fig. S1.

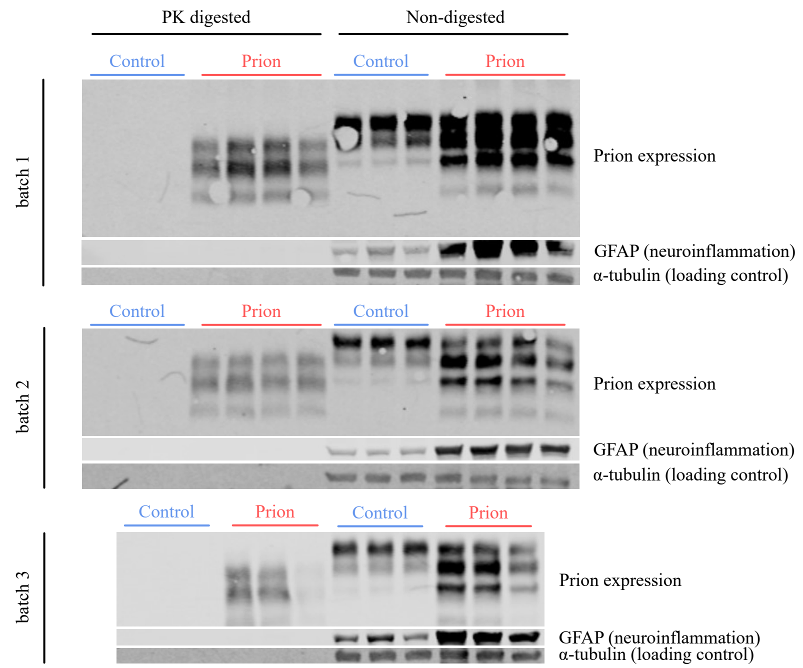

Fig. S1. Raw Western blots. Western blots showing prion protein and GFAP expression for the prion-tg37 (11 samples) and control-tg37 (9 samples) brain slices. Blots of α-tubulin, a loading control, is also shown. The misfolded prion protein expression can be determined from the PK digested columns and the GFAP and α-tubulin expression from the non-digested columns.
